# Neural correlates and sex hormonal associations of impaired feedback-learning in adolescent patients with anorexia nervosa - a longitudinal fMRI study

**DOI:** 10.1016/j.ynirp.2026.100408

**Published:** 2026-09-30

**Authors:** Kimberly Büttgen, Jana Goedecke, Lukas Stanetzky, Brigitte Dahmen, Beate Herpertz-Dahlmann, Kerstin Konrad, Oezguer A Onur, Jochen Seitz

**Affiliations:** a Department of Child and Adolescent Psychiatry, Psychosomatics and Psychotherapy, RWTH University Hospital Aachen, Neuenhofer Weg 21, Aachen, 52074, Germany; b Department of Neurology, University Hospital Cologne, Kerpener Straße 62, Köln, 50937, Germany; c Institute for Medical Psychology and Medical Sociology, RWTH Aachen University, Pauwelsstraße 19, Aachen, 52074, Germany; d Institute of Neuroscience and Medicine, Translational Neuroscience (INM-10), Research Centre Juelich, Juelich, Germany; e Department of Child and Adolescent Psychiatry, Psychosomatics and Psychotherapy, LVR University Hospital Essen, Wickenburgstr. 21, Essen, 45147, Germany

**Keywords:** Lingual gyrus, Fusiform gyrus, Thalamus, Learning, Eating disorder, Neuroimaging, Adolescents

## Abstract

**Introduction:**

Anorexia nervosa (AN) is a somato-psychiatric disease that mostly begins in adolescence, involving endocrine changes including amenorrhea. It is difficult to treat as most patients have difficulties embracing change and thus, the disease often takes a chronic course. Underlying pathomechanisms including moderate neuropsychological deficits of learning and memory remain insufficiently understood. As learning and memory have been shown to be estradiol-dependent in healthy controls (HC), we conducted this assessment to investigate the impact of reduced estradiol in patients on verbal learning, memory and the underlying brain-alterations.

**Methods:**

We studied feedback-learning in 20 adolescent patients with AN at admission and following short-term weight restoration, compared to 26 age- and sex-matched HC using fMRI. Learning performance and brain alterations were compared between groups and longitudinally within the patients and their interdependence with sex hormone levels as well as clinical parameters was explored.

**Results:**

Reduced brain activity at admission could be shown in left occipital as well as calcarine, lingual and fusiform gyrus, Ncl. caudatus and thalamus compared to HC. Following weight-restoration, activity increased along with patients’ learning rate. Exploratory analyses showed brain activations to be relatively higher with shorter illness duration, its improvement was significantly greater in less starved patients. Estradiol levels were associated with learning in HC but not in patients.

**Discussion:**

Feedback-learning appears deteriorated in the acute state of AN. Neural hypoactivity might make re-learning and psychotherapy more difficult thus requiring adequate renourishment first. No direct link to sex hormone-reduction in patients could be shown in this study.

## Highlights

• Patients with AN show deficits in learning but pathomechanisms remain unclear.
• We studied learning and its association with estradiol in a longitudinal fMRI-study.
• We found reduced activity in fusiform gyrus, ncl. caudatus and thalamus in AN.
• Neural activity increased alongside improving learning ability after weight gain.
• Early weight rehabilitation might thus facilitate re-learning in AN-psychotherapy.

## 1 Introduction

Anorexia nervosa (AN) is a severe somato-psychiatric disease, with onset predominantly in adolescence. It is characterized by insufficient nutritional intake, body image disturbances and fear of weight gain as well as physiological changes including amenorrhea and reduction of estradiol(Herpertz et al., 2019). Diagnostic criteria (following DSM-5) include a BMI under the 10th age-related percentile that cannot be explained by another health condition or the unavailability of food or alternative a weight loss of at least 20% in the last 6 months. Furthermore, behavioral changes can be seen that lead to the weight-loss, such as restricted eating, purging (i.e. vomiting or use of laxatives) and excessive exercise. These different patterns lead to a subdivision into different types of anorexia. What they all have in common as a diagnostic criterion is the fear of weight-gain. (Association, 2013; World Health Organization, 2019). A low BMI, and thus the extent of starvation, correlates with increased mortality and can therefore be used as a surrogate parameter for the severity of the disease and thereby as an indicator of disease activity (Herpertz-Dahlmann, 2015).

In adolescence, eating disorders are among the most common illnesses that lead to a high number of disability-adjusted life years. Anorexia nervosa is known as one of the psychiatric disorders with the highest mortalities (Herpertz-Dahlmann, 2021). Multimodal treatment of AN encompasses nutritional therapy and weight restoration as well as psychotherapy. There are currently no approved drugs for the treatment of AN. It is notoriously difficult to treat as most patients are afraid of gaining weight, have difficulties embracing suggestions made in psychotherapy and appear rigid. AN often takes a chronic course, with only around 50% of patients after 7 years completely remitting (Solmi et al., 2024). AN is also known to have a long lasting impact not only on the hormonal status of the patients, especially shown in persistent hypoestrogenism, but also affect the gastrointestinal, skeletal and cardiovascular system, and thus should be diagnosed and treated as soon as possible (Meczekalski et al., 2013). Peak onset is during the period of puberty, and AN in adolescent patients needs to be treated quickly to prevent lasting changes to bone metabolism, growth and changes within the physiological course of brain alterations, known to be relevant until early adulthood (Olivo et al., 2019). Investigating brain alterations in adolescent patients with AN furthermore reduces confounding by adaptation processes during long illness-duration and potential treatment effects. It shows the effects of starvation in a neuroendocrinological critical window and even targets a phase, where early feedback-learning can predict weight-gain outcome (Frank et al., 2018).

Neuropsychological deficits in patients with AN are well studied on a behavioral level. Stedal et al. included 1333 participants in a meta-analysis (665 with AN) that showed small but significant impairments in decision making, central coherence and visuospatial abilities, as well as theory of mind, memory and working memory (Stedal et al., 2021). Especially the latter impairments might affect psychotherapy as it makes learning and relearning, typical elements of successful psychotherapy leading to modifications of behaviour, more difficult.

Former feedback-learning studies have shown that feedback learning in healthy individuals can be subdivided in two underlying principal mechanisms, driven by reward or punishment and associative or category-learning. The first mainly activates ventral-striatal and medial-frontal circuits, whilst in the latter, successful learning is positively correlated with activation of caudate nucleus, and negatively with activation in hippocampus (Reavis et al., 2015; Seger and Cincotta, 2005). In adolescent AN, reduced learning from positive feedback could be shown (Uniacke et al., 2024).

Underlying pathomechanisms of these neuropsychological deficits of learning and memory in AN, especially according to sex-hormon levels remain unclear. Existing and partly contradicting evidence points to a potential role of multiple brain regions including the dorsal anterior cingulate cortex (dACC) (Geisler et al., 2017), the dorsolateral prefrontal cortex and the striatum (Ehrlich et al., 2015; Lao-Kaim et al., 2015), the thalamus (Firk et al., 2015) and the hippocampus (Keeler et al., 2020). Especially longitudinal studies in adolescents are scarce but could give important insights into the role of starvation and could disentangle state-from trait-effects (Brown et al., 2024). Another notable aspect in this context is that learning and memory have been shown to be among other estradiol-dependent in healthy humans (Hemmingsen et al., 2021; Taxier et al., 2020) and animal models of AN (V. Luine, 2015; Paulukat et al., 2016). Whilst estrogen can e.g. increase memory consolidation in an animal model after administration within 1 h through the increased expression of membrane-bound estrogen receptors, it furthermore regulates the spinogenesis in the hippocampus (V. N. Luine and Frankfurt, 2012; Prange-Kiel and Rune, 2006). Estrogens thus influence cognitive function through the modulation of neuronal structures with implications for the treatment of a range of brain disorders, for example schizophrenia and depression (Sellers et al., 2015). In patients with AN, known to lack estradiol alongside widespread amenorrhea, associations of reduced estradiol and memory have rarely been assessed. Verbal learning was positively correlated with estradiol in acutely ill adolescent patients (Buehren et al., 2011), while recognition-learning was estradiol associated in the activity-based anorexia animal model in starved animals, a significant correlation between lowered estradiol-levels and impaired object recognition memory could be shown (Paulukat et al., 2016). Yet, hormonal replacement with estradiol in patients with AN should, according to the German S3 treatment guidelines for AN for the compensation of accompanying osteoporosis, be critically considered if patients have been suffering from severe AN for several years (Herpertz et al., 2019). Thus, finding a link between estradiol levels and learning could have important therapeutic consequences by extending this indication of estradiol substitution to learning difficulties. To our knowledge, no neuroimaging studies have yet attempted to study the influence of sexual hormone levels on the memory function and learning in patients with AN.

We thus set out to fill this gap using a well-established feedback-learning task in a longitudinal design in adolescent patients with AN before and after short-term weight recovery. Our hypotheses were to find moderately decreased learning rates in patients with acute AN compared to healthy controls (HC) that increased with weight gain. We suspected hippocampal, parahippocampal and thalamic regions to show hypoactivation associated with this reduced learning. Lastly, we hypothesized estradiol-levels to be positively associated with learning rate and neural activity.

## 2 Material and method

### 2.1 Participants

Twenty-two female adolescent patients at the age of ten to eighteen, inpatients at the Department of Child and Adolescent Psychiatry of Aachen University Hospital, with a diagnosis of AN based on the DSM-5 criteria (American Psychiatric Association, 2013), and twenty-eight healthy female age-matched HCs were initially included in this study. When recruiting the HC via ads and flyers, the focus of this study (i.e. Anorexia nervosa) was not disclosed to prevent any possible bias when selecting the HC. All subjects and their legal representatives gave written informed consent. All experiments including data collection, assessment and analysis were conducted following a standardized protocol to minimize bias. Especially MRI-analysis, neuropsychological test evaluation and questionnaire analysis followed the same objective and automated protocol for patients and healthy controls alike. The study was carried out in accordance with the Declaration of Helsinki and approved by the local ethics committee (Ethics Committee of the RWTH Aachen Faculty of Medicine, EK081/10). Treatment at the eating-disorder department was composed of a multimodal and multidisciplinary program following the German S3-guidelines (Herpertz et al., 2019) including weight restoration, cognitive-behavioral and family oriented therapy.

### 2.2 Clinical assessments

At the time of admission, the patients met the criteria for AN according to the DSM-5 as determined using the Eating disorder examination expert interview (EDE) (Hilbert et al., 2004). Weight was quantified by age- and sex-adjusted body mass-index standard deviation score (BMI-SDS), calculated according to Kromeyer-Hauschild et al. (2001) and had to be lower than the 10th age related percentile for inclusion. Eating disorder symptoms were quantified using the Eating Disorder Inventory-2 (EDI-2) (Kappel et al., 2012; Paul and Thiel, 2005), depressive symptoms were evaluated by the Beck Depression Inventory (BDI) (Hautzinger et al., 2006), and anxiety symptoms via the Spence Children's anxiety Scale (SCAS) (Spence, 2012). The following exclusion criteria for patients were applied: history of psychosis, severe substance abuse, IQ < 80 and a weight above the 10th age related percentile. 4 Patients were under medication of olanzapin whilst undergoing the first MRI-scan, one of them also took Lorazepam on a regular basis. 6 patients were under psychotropic medication during the second MRI-scan, including atypical neuroleptics (olanzapine n = 4, risperidone n = 1) selective serotonine reuptake inhibitors such as Fluoxetin (n = 1) and Sertralin (n = 2). The medication corresponded to the dosage recommendation for the corresponding age group. All of the patients received calcium substitution whilst being inpatient.

Inclusion criteria for HCs were: no psychiatric diagnoses and no drug intake, weight above the 10th age related percentile (BMI-SDS), below the cut-off in psychometric testing for eating disorders and depression (EDI-2 and BDI-2, see below) and no use of hormonal contraception. All subjects had to meet the in-house criteria for MRI (e.g. no metal implants in the body etc.).

The interviews were performed by trained and supervised clinical researchers. Four subjects were excluded post-hoc, two of them were patients. One patient was excluded due to temporary disruption of inpatient treatment, another due to missing responses in >40% of the events during the fMRI-experiment. Two HCs were excluded, one due to poor quality of the MRI images and the other one because of excessive head movement (see 2.4.2). Ultimately, twenty patients with AN and twenty-six HC formed the sample of the study. Missing values were excluded from the respective analysis. The demographical and clinical characteristics can be found in Table 1.

**Table 1:** Clinical characteristics, hormones and learning tests.

	AN_Admission_ N = 20 mean (SD)	AN_Discharge_ N = 20 mean (SD)	HC N = 26 mean (SD)	AN_Admission_ vs. HC p	AN_Discharge_ vs. HC p	AN_Admission_ vs. AN_Discharge_ p
**Clinical characteristics**						
**Age**, years	15.22 (2.05)	15.53 (2.01)	15.78 (2.00)	0.350	0.676	<0.001
*N/df*	*20*	*20*	*26*	*44*	*44*	*19*
**BMI,** kg/m^2^	15.72 (1.52)	18.15 (1.19)	22.29 (3.05)	<0.001	<0.001	<0.001
*N/df*	*20*	*20*	*26*	*44*	*44*	*19*
**BMI-SDS**	−2.26 (1.14)	−0.90 (0.40)	0.50 (0.91)	<0.001	<0.001	<0.001
*N/df*	*20*	*20*	*26*	*44*	*44*	*19*
**Weight loss**, BMI-SDS	−2.82 (0.94)					
*N*	*20*					
**Illness duration**, weeks	71.50 (65.35)					
*N*	*20*					

**EDI-I**I	264.30 (99.76)	223.01 (98.48)	192.58 (42.23)	0.002	0.167	0.174
*N/df*	*20*	*18*	*26*	*44*	*42*	*17*
**BDI-II**	19.29 (14.11)	13.50 (14.18)	4.50 (5.29)	<0.001	0.005	0.183
*N/df*	*17*	*18*	*26*	*41*	*42*	*15*
**SCAS**	26.17 (18.17)	20.56 (16.04)	13.46 (7.17)	0.010	0.093	0.326
*N/df*	*18*	*18*	*26*	*42*	*42*	*16*
**Hormones**						
**LH** U/l	2.29 (4.60)	7.09 (4.27)				0.032
*N/df*	*19*	*12*				*10*
**FSH** IU/l	4.24 (4.53)	5.85 (1.91)				0.868
*N/df*	*17*	*12*				*9*
**Estradiol** pg/ml	56.10 (21.3)	70.30 (28.50)	117.16 (99.40)	0.01	0.053	0.056
*N/df*	*20*	*19*	*26*	*44*	*43*	*18*
**Medication**						
**Atypical neuroleptic**	4	5	0			
**SSRI**	0	3	0			
**Benzodiazepine**	1	0	0			
**Learning tests**						
**AVLT**, learning	56.25 (7.48)	58.58 (8.20)	57.76 (11.98)	0.625	0.390	0.257
*N/df*	*20*	*19*	*25*	*43*	*42*	*18*
**AVLT**, free recall	12.10 (2.51)	12.95 (1.50)	12.48 (1.83)	0.560	0.757	0.263
*N/df*	*20*	*20*	*19*	*43*	*42*	*19*
**AVLT**, delayed recall	11.55 (2.63)	12.22 (2.53)	12.40 (2.86)	0.305	0.414	0.286
*N/df*	*20*	*18*	*25*	*43*	*41*	*17*
**AVLT**, word recognition	14.55 (0.76)	14.28 (0.89)	14.48 (1.12)	0.813	0.531	0.386
*N/df*	*20*	*18*	*25*	*43*	*41*	*17*
**RFT,** Sum correct recognitions	38.50 (7.99)		43.48 (9.46)	0.025		
*N/df*	*20*		*23*	*41*		
**RFT,** Sum false positives	11.05 (8.81)		8.09 (7.45)	0.007		
*N/df*	*20*		*23*	*41*		
**RFT**, Sum difference correct-false positives	27.45 (9.69)		35.39 (8.35)	0.003		
*N/df*	*20*		*23*	*41*		
**RFT**, lability index, mean	0.32 (0.28)		0.42 (0.31)	0.301		
*N/df*	*20*		*23*	*41*		

Values are reported as the mean and standard deviation in brackets, and maximum and minimum values in square brackets. Abbreviations: AN: Anorexia nervosa, HC: Healthy controls, SD.: standard deviation; BMI: Body Mass Index; BMI-SDS: Standardised age adjusted BMI; Weight loss: Weight that was lost prior to admission; SSRI: Selective serotonine reuptake inhibitor; EDI-II: Eating Disorder Inventory-II; BDI-II: Beck's Depression Inventory II; SCAS: Spence's Child and Adolescent Anxiety Scale. AVLT: Rey Auditory-Verbal Learning and Memory test; learning: sum of the learned words; free recall: sum of the remembered words after different words; delayed recall: sum of remembered words after 20 min; word recognition: correct answers whether words are known or not; RFT: Recurring Figures Test; Sum correct recognitions: Sum of the correct answers; Sum false positive: Sum of the incorrect answers; Sum diff: Sum of the difference of correct recognitions and false positives; lability index: change in between the sections. Student's T-Tests were used to calculated group differences. Missing values were excluded from the respective analysis.

**Table 2:** Significant clusters for the HC > AN_Admission_ (Hits > False) contrast with anatomical localization and statistical parameters.

	Anatomic region	Cluster FWE	Voxel	Peak-T	MNI coordinates (x, y, z)
1.	Cluster covering parts of left middle and inferior occipital gyrus as well as left fusiform gyrus and lingual gyrus	0.000	273	6.48	−36, −75, 0
2.	Cluster covering parts of left middle and superior occipital gyrus as well as left calcarine and lingual gyrus	0.000	314	5.59	−18, −90, 9
3.	Cluster in left thalamus & caudate	0.001	82	5.14	−12, −9, 12

**Table 3:** Significant clusters for the AN_Discharge_ > AN_Admission_, Hits > False contrast with anatomical localization and statistical parameters.

	Anatomic region	Cluster FWE	Voxel	Peak-T	MNI coordinates (x, y, z)
4.	Cluster covering parts of left calcarine and lingual gyrus as well as left middle and superior occipital gyrus	0.000	113	5.56	−9, −90, 6
5.	Cluster covering parts of lingual gyrus as well as fusiform gyrus while a small part of this cluster ranges into the cerebellum	0.026	51	5.08	−15, −78, −24

All subjects underwent neuropsychological testing at admission and discharge or, in case of the HC, at their appointment for the MRI. In addition to the paradigm performed in the MRI, the learning ability was determined by means of two clinical tests, assessing the verbal and nonverbal learning abilities. German adaptations of the Kimura's recurring-figures test (Hartje and Rixecker, 1978; Rixecker and Hartje, 1980) (RFT), the “Nonverbaler Lerntest” (Kimura, 1963) and Rey auditory-verbal learning and memory test (AVLT), the “Verbaler Lern-und Merkfähigkeitstest” (Müller et al., 1997) were used. For better readability, the original English names are used to describe the tests. As these were age-matched samples, raw values were compared.

#### 2.2.1 EDI-II

The Eating Disorder Inventory-2 (Garner et al., 1983)German translation is a multidimensional self-report questionnaire for assessing disordered eating behavior and associated psychological characteristics.

#### 2.2.2 BDI-II-R

To asses depressive symptoms, the German translation of the Beck's Depression Inventory II-Revised (Hautzinger et al., 2006; Kühner et al., 2007) containing 21 questions was used.

#### 2.2.3 Kimura's recurring-figures test (RFT)

The German adaptation of the RFT by Sturm and Willmes (Sturm and Willmes, 1999) consists of 160 geometric or nonsense figures on small cards that are presented consecutively for 3 s to the subjects. Eight of the figures are shown seven times each (stimulus figures), the other (new) figures do not repeat. The subject decides for each card shown whether it is known or unknown. To evaluate learning, correct answers (RFT, sum correct recognitions), false positive answers (RFT sum false positives) and the difference (RFT sum difference) were evaluated for all seven rounds separately (Rixecker and Hartje, 1980). As a measure of the stability of learning performance, all blocks were considered and the lability index was calculated by measuring whether each item was remembered correctly or incorrectly in a consistent manner (RFT, lability index). The RFT was only performed at AN_Admission_ and in HC as there is only one version of the test.

#### 2.2.4 Rey auditory-verbal learning and memory test (AVLT)

The AVLT (Helmstaedter and Durwen, 1990) evaluates verbal memory via the subject learning a list of 15 independent words and its immediate retrieval, retrieval after distraction, retrieval after a break of 30 min and recognition of the words. There are two different versions of the word-list (Helmstaedter and Durwen, 1990). The subjects were randomly assigned to a version and completed the other version at the second timepoint to avoid habituation effects. The execution of the test consists of an auditory presentation of the word list and the instruction to repeat as many words as remembered. This procedure is repeated five times, the sum of the right positive nominations is called “learning” and represents the short-time component of memory. After that, another list, consisting of 30 different words is presented and must be learned. The retrieval of the first word list after that (two times via “free recall”) shows the long term memory-component indifferent to interference learning. After a break of 30 min, another retrieval is asked for (Müller et al., 1997). After that, a list of 50 words, including each word from the two lists plus 20 independent words is presented, and the subject is asked whether the word is known and to which wordlist it belongs (“word recognition”).

### 2.3 Sex hormone analysis

Blood samples were taken at admission and discharge and from the healthy controls after a morning fast. The samples were immediately centrifuged and frozen at −80° until they were analyzed for Estradiol ELISA (EIA-2693, DRG Instruments). Luteinizing hormone (LH) and follicle stimulating hormone (FSH) were analyzed using the clinical hospital routine using Elecsys ECLISA (cobas) (IRP 78/549; NIBSC 80/552).

### 2.4 Imaging paradigm: item-category association task

Patients with AN were measured twice in the MRI, once after admission (AN_Admission_) and second time at discharge with a target weight near the 25th BMI percentile in combination with clinical signs of recovery (AN_discharge_). Before each measurement, the paradigm was explained to the subjects, and they had the opportunity to practice the procedure outside the MRI. The measurements were planned to suit the normal inpatient daily routines in order to not affect their scheduled meals or therapies. The control group (HC) was measured once.

The implicit learning paradigm in the scanner was adapted from previous studies on adults (Onur et al., 2010). Participants were instructed to push a button of a remote response box (Button Boxes (LUMItouch by Photon Control Inc., Burnaby, BC, Canada) in each hand with their index fingers after the presentation of a three-digit numerical item. They were told that there was a random algorithm defining which item belonged to category ‘A’ or ‘B’. Once assigned, categories remained unchanged over six presentations (cycles). After each guess, there was visual feedback which indicated if the response was correct or incorrect. No response after 3 s resulted in no feedback and the event was recorded as a ‘miss’. The feedback informed the subjects about the correct category and enabled them to progressively learn and improve their performance over consecutive cycles. Each participant completed four runs in which eight items were presented in six cycles, therefore the number of events per run was 48, leading to 192 events over the entire paradigm. Items were presented in a random order within each cycle. The trial duration was 3500 msec, separated in a stimulus-response duration of 2500 msec and a feedback duration of 1000 msec, jittered by an intertrial interval of 2250 msec (1500 – 3000 msec). Total scanning time of our paradigm was 20 min, Goggles were used for stimulus presentation and Presentation12 (Neurobehavioral Systems, Inc, Albany, California) for carrying out stimulus delivery and response recording. Possible answers of ‘A’ or ‘B’ appeared on the screen either on the left or the right side of the three-digit number determining the corresponding button the participants had to push. Lateralization was randomly changed to prevent simple visuomotor learning.

### 2.5 Acquisition of imaging data

A TIM Trio MRI System (Siemens, Erlangen, Germany) operating at 3T was used to acquire T2*-weighted echo planar images with blood oxygenation level-dependent (BOLD) contrast. Repetition time was 2.0 s; echo time 28 msec, matrix size 64 x 64; pixel size 3.0 x 3.0; slice thickness 3.5 mm; distance factor 10%; field of view 192 mm; flip angle 77°; 34 axial slices were acquired in 156 volumes. A total of 5 fMRI sequences were performed as part of the paradigm. These lasted 4 × 5.8 min and 10.4 min.

To stabilize the magnetic field, we discarded the first four volumes. We also acquired high-resolution anatomical magnetic resonance images (T1 weighted, three-dimensional MP-RAGE (magnetization-prepared rapid acquisition with gradient echo)) for registration purposes.

The parameters for the anatomical scans were as follows: TE, 3.03 ms; TR, 1880 ms; flip angle, 9°; FoV, 256 mm × 256 mm; and slice thickness 1 mm, 176 slices were acquired for whole brain coverage. The acquisition was ascending interleaved.

### 2.6 Analysis

#### 2.6.1 Analysis of clinical and behavioral data

Clinical and behavioral data were analyzed using SPSS, version 25.0.0.1 (IBM, Armonk; N.Y.). All parameters were tested for normal-distribution using the Shapiro-Wilk test. Normally distributed variables were compared using Student-T-tests, in case of a violation of normal distribution, non-parametric Mann-Whitney-U tests were applied. We compared patients at admission and discharge with HC using independent t-tests and patients at admission with patients at discharge using paired t-tests, respectively.

“Performance” of the in-scanner task was calculated by building a mean of correct answers over all four sessions. Additionally, we calculated the learning rate defined as the increase in correct answers compared to the initial trial (with about but not exactly 50:50 correct:incorrect due differential initial guessing). T-Tests and Mann-Whitney U Tests were used to compare performance, while repeated-measures ANOVAs were calculated to compare increasing learning rates (HC vs. AN_Admission_, HC vs. AN_Discharge_ and AN_Admission_ vs. AN_Discharge_).

##### 2.6.1.1 Exploratory analysis of associations of learning with clinical parameters and sex hormone levels

Correlation analyses were calculated to test for associations between learning parameters outside (Kimura's Recurring-Figures Test RFT: Total learning, interference learning, delayed recall and Rey's Auditory-Verbal Learning test AVLT: Total correct, total incorrect and total difference) and inside the scanner (performance and learning rate) with the clinical parameters BMI-SDS and illness duration and with the sex hormones estradiol, LH and FSH for all three comparisons (HC vs. AN_Admission_, HC vs. AN_Discharge_ and AN_Admission_ vs. AN_Discharge_) using Pearson's and Spearman's correlations where appropriate.

#### 2.6.2 Analysis of imaging data

Preprocessing and analysis of imaging data was performed using DPARSF 4.3 (data preprocessing assistant for resting state fMRI), part of DPABI V3.0 (Data processing and analysis for brain imaging, a MATLAB Toolbox mainly based on SPM 12 (statistical parametric mapping, https://www.fil.ion.ucl.ac.uk/spm/software/spm12/) (Yan et al., 2016). Any head motion effects were regressed from the realigned data by utilization of the Friston 24-parameter model (Friston et al., 1996). The Jenkinson definition of the mean framewise displacement (FD) (Jenkinson et al., 2002) was used to detect individuals that had to be excluded due to excessive movement. The cut-off applied was more than two times over the standard deviation of the sample's mean FD (Jenkinson), which led to the exclusion of one HC, as mentioned in 2.1. Then, structural images were coregistered to the mean functional images and segmented into grey matter, white matter and CSF. The DARTEL tool (Ashburner, 2007) was used to transform the structural images (T1-weighted MPRAGE) into the MNI space (Collins et al., 1994; Holmes et al., 1998) and the resulting parameters were used to spatially normalize the functional images. Images were resampled at a voxel size of 3 x 3 × 3 mm and spatially smoothed using an 8-mm full-width at half maximum Gaussian kernel.

We defined a regressor that indicated the onset times of all trials recording a desired event (hit, false or miss). The hemodynamic response was modelled using a canonical hemodynamic response function (HRF) and its first derivative, which included the six head movement parameters as confounds. For our first-level analysis we calculated a linear baseline contrast comparing the regressors with the implicit baseline. This contrast was then taken to the second level, where it was subjected to two-sample or paired-sample T-Tests and bidirectional contrasts were calculated.

To test the neural correlate of (increasingly) correct recall and its associated neural correlates, we calculated the difference between correct hits and false answers, leaving out misses. We calculated whole brain analyses for all three contrasts (HC vs. AN_Admission_, HC vs. AN_Discharge_ and AN_Admission_ vs. AN_Discharge_). Significant results are reported at an FWE-corrected cluster threshold of *p* < 0.05 (cluster forming threshold *p* < 0.001). The feasibility of this approach has been confirmed by our previous work (Goossens et al., 2009; Hurlemann et al., 2008; Onur et al., 2009).

For anatomical labelling, we have used MRIcroGL and aal as cited in the acknowledgments. All the included structures in the clusters can be found in Supplementary Table 1.

#### 2.6.3 Exploratory analysis of associations of brain activations with clinical parameters, learning and hormone levels

To test for brain-behavior response relationships, we extracted the beta values of all significantly different clusters yielded by the group comparisons by averaging neural activations over all contained voxels per cluster. Associations between beta values of neural activation and the clinical parameters BMI-SDS and illness duration, the learning parameters outside (RFT: total learning, interference learning, delayed recall and AVLT: Total correct, total incorrect and total difference) and inside the scanner (performance and learning rate) and with the sex hormones estradiol, LH and FSH were calculated for the three comparisons (HC vs. AN_Admission_, HC vs. AN_Discharge_ and AN_Admission_ vs. AN_Discharge_).

## 3 Results

### 3.1 Analysis of clinical and behavioral data

#### 3.1.1 Group differences

AN and HC did not differ significantly in age. As expected, AN at both timepoints and HC differed significantly in BMI SDS, EDI-2, BDI-2, SCAS (all *p* < 0.05, Table 1).

AN_Admission_ showed a trend to a lower learning rate in the Kimura's Recurring-Figures Test (RFT) *t*-test for “sum correct recognitions” (*t*(41) = 1,864, *p* = 0.069) and a significantly reduced “difference correct-incorrect”, (*t*(41) = 2,887, *p* = 0.006) compared to HC.

Rey's Auditory-Verbal Learning Test AVLT showed nominally lower but insignificantly different values in AN_Admission_ compared to HC and no differences in AN_Discharge_ nor longitudinally.

No significant differences in in-scanner performance between groups were observed (Fig. 1).

**Fig. 1:**
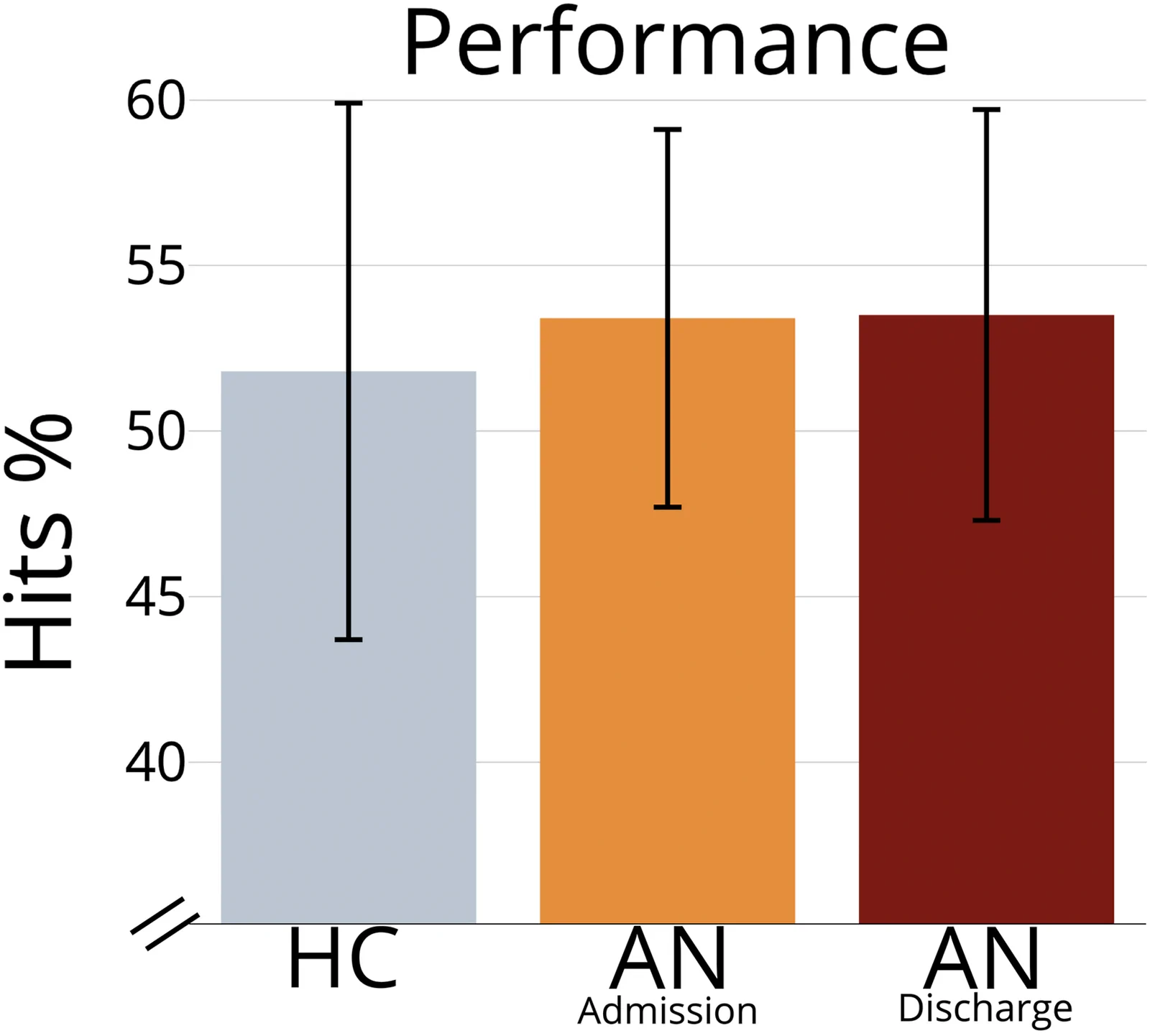
In-scanner performance over the entire paradigm per group, mean and standard deviation in % of correct hits during the learning task.

However, learning rate increased significantly from AN_Admission_ to AN_Discharge_ (*t*(19) = 2.508, *p* = 0.021), reflecting higher values at discharge. Moreover, learning rate was higher in AN_Discharge_ compared to HC (*t*(44) = 2.775, *p* = 0.008), noting that positive t-values reflect the direction of the underlying contrast. (Fig. 2).

**Fig. 2:**
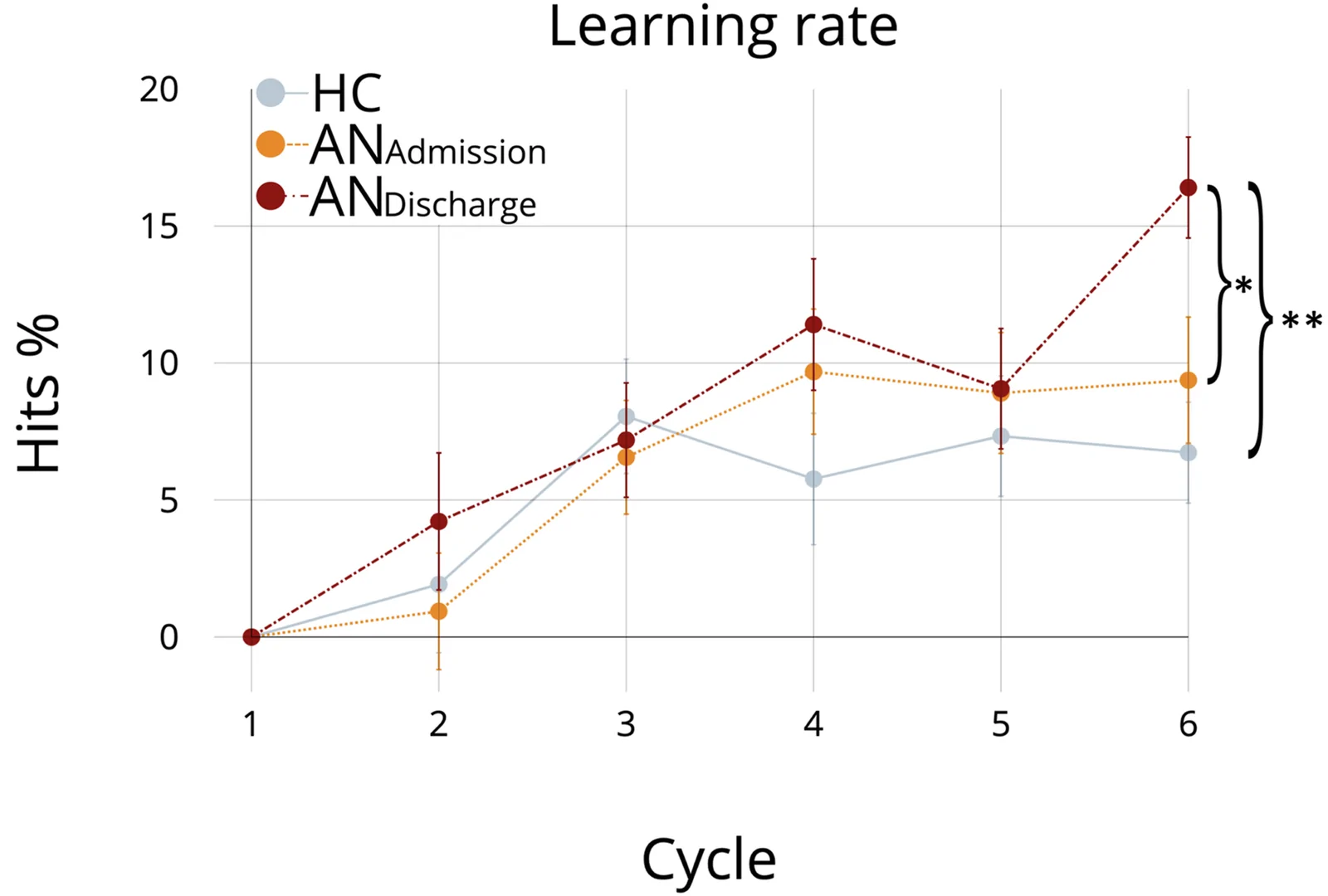
Learning rate in-scanner per cycle per group, calculate as performance in cycle 6 - performance in cycle 1 with standard error. *p < 0.05 ** < 0.01 in paired/unpaired student's T-Test respectively.

#### 3.1.2 Association of learning with clinical parameters

In-scanner performance was associated with out-of-scanner total recall *r* = 0.511, *p* = 0.021 and delayed recall in the *r* = 0.488, *p* = 0.029 in the Kimura's recurring-figures test in AN_Admission_ further validating the in-scanner measurements (supplementary figure 2A).

We did not find a significant association of estradiol with in-scanner learning rate in AN and only trend-level for HC r = 0.355, p = 0.075). As previous research had found an influence of estradiol on learning in HC and patients with AN, we performed a median split on HC as well as patients with AN at admission and discharge in upper and lower halves using serum estradiol levels and compared their in-scanner learning rates and performances separately. A trend for a group difference was found for AN_Discharge_ (p = 0.105) for in scanner performance (Supplementary Fig. 3) and there was a significant association of in-scanner learning rate with estradiol in the higher-estradiol HC *r* = 0.664, *p* = 0.013, n = 13 but not in patients with AN (AN_Admission_ r = 0.093, p = 0.799, AN_Discharge_ r = 0.416, p = 0.265; Supplementary Fig. 2B).

### 3.2 Analysis of imaging data

Whole brain analysis comparing AN_Admission_ and HC showed significant hypoactivation in AN_Admission_ in three significant clusters in left superior, middle and inferior occipital gyrus as well as calcarine gyrus, lingual and fusiform gyrus, Ncl. caudatus/thalamus compared to HC (Fig. 3). Whole brain analysis comparing AN_Discharge_ with AN_Admission_ resulted in two very similarly located significant clusters showing increasing activation in calcarine, lingual and fusiform gyrus as well as middle and superior occiptal gyrus while a small part of one cluster ranges into the cerebellum (Fig. 4). Extracted beta-plots of all five significant clusters revealed lower activation in AN_Admission_ to approach levels of HC in AN_Discharge._ in all clusters (Fig. 3 + 4).

**Fig. 3:**
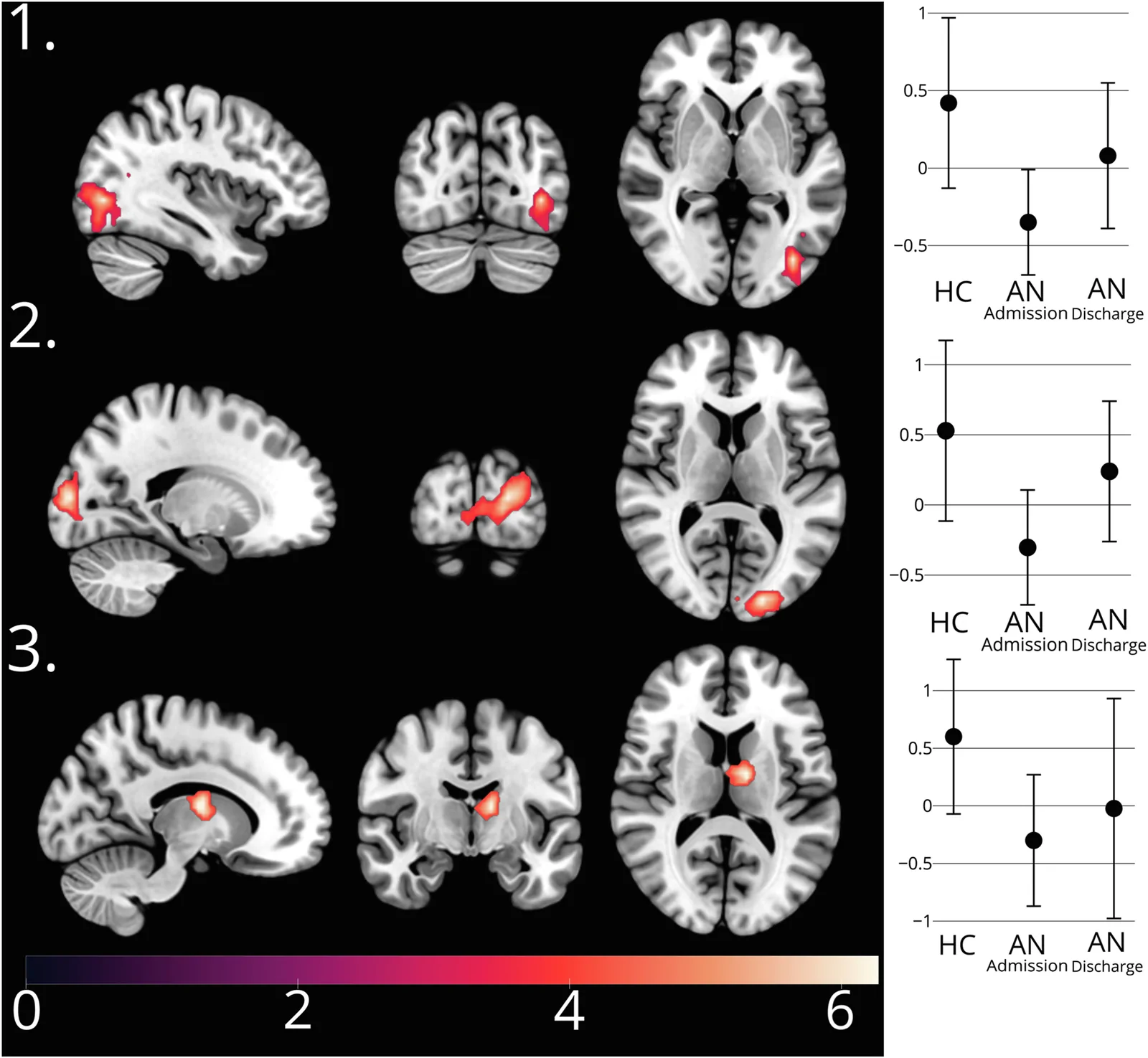
HC > AN_Admission_, Hits > False Clusters significant after FWE correction; here 0.001 uncorr. for display purpose only, for details see Table 2. Plots on the right show mean extracted β estimates from the significant clusters; error bars represent 95% confidence intervals.

**Fig. 4:**
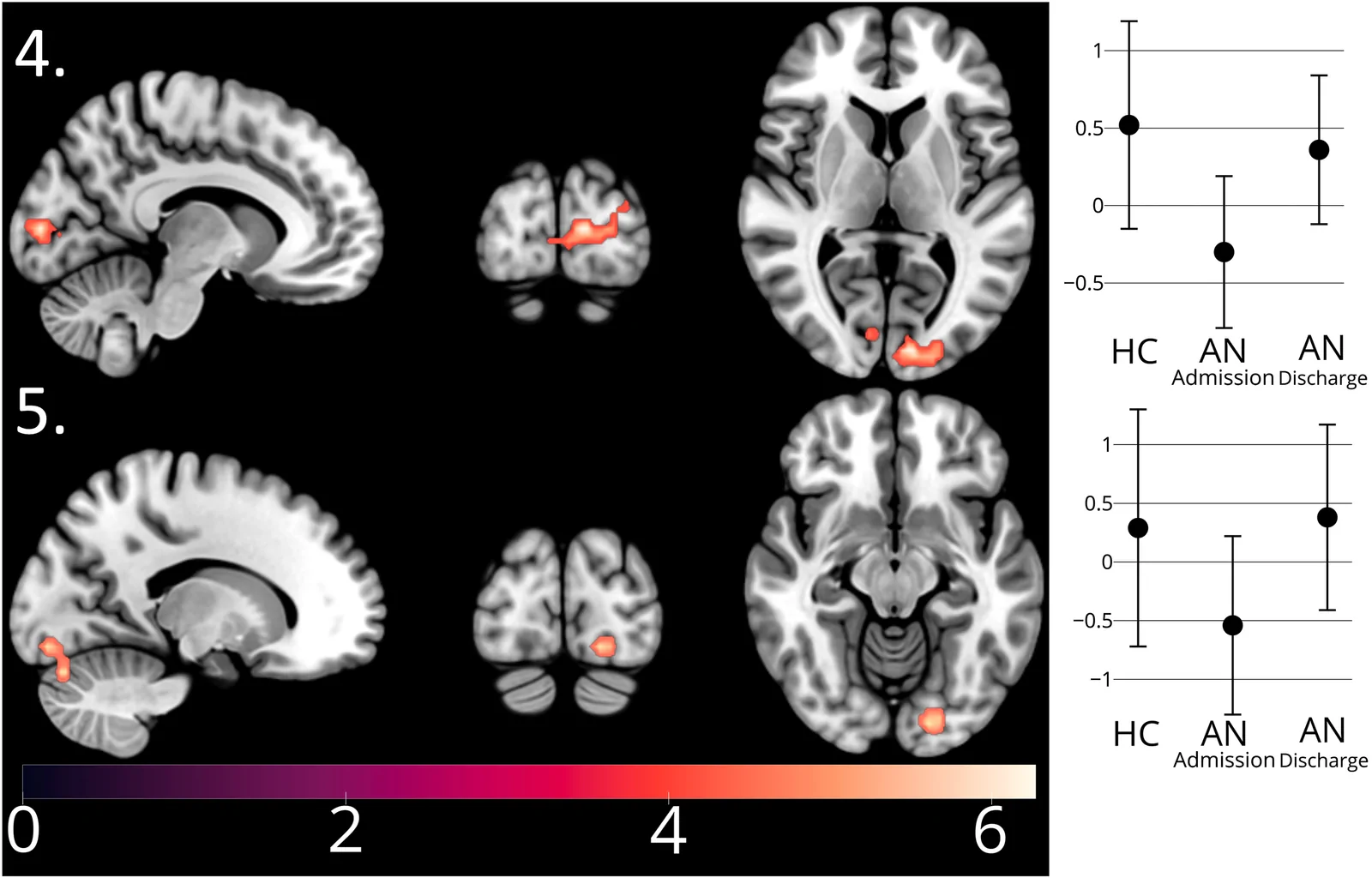
AN_Discharge_ > AN_Admission_, Hits > False, Clusters significant after FWE correction; here 0.001 uncorr. for display purpose only, for details see Table 3. Plots on the right show mean extracted β estimates from the significant clusters; error bars represent 95% confidence intervals.

Subanalyses of beta plots split in betas of neural signatures of hits and false revealed that the observed pattern in the second level analysis is reflected in the analysis of the betas in hits rather than in false. (Supplementary Fig. 1).

No significant differences persisted between AN_Discharge_ and HC.

### 3.3 Exploratory analysis of associations of brain activations with clinical parameters, learning and hormone levels

Brain hypoactivation based on the contrast AN_Admission_ < HC showed no positive correlations between Cluster 2 activation (Cluster covering parts of left middle and inferior occipital gyrus as well as left fusiform gyrus and lingual gyrus) and delayed recall but demonstrated a positive correlation between difference in Cluster 2 activation and BMI-SDS at admission (Fig. 5 A). A negative correlation was found between difference in Cluster 2 activation and BMI-SDS at admission and future LH increase (Fig. 5A). There was a negative correlation between Cluster 1 activation and duration of illness (Fig. 5B). In the thalamus and caudate there was a negative correlation between the difference in activation (AN_Discharge_- AN_Admission_) and future LH increase (Fig. 5C).

**Fig. 5A:**
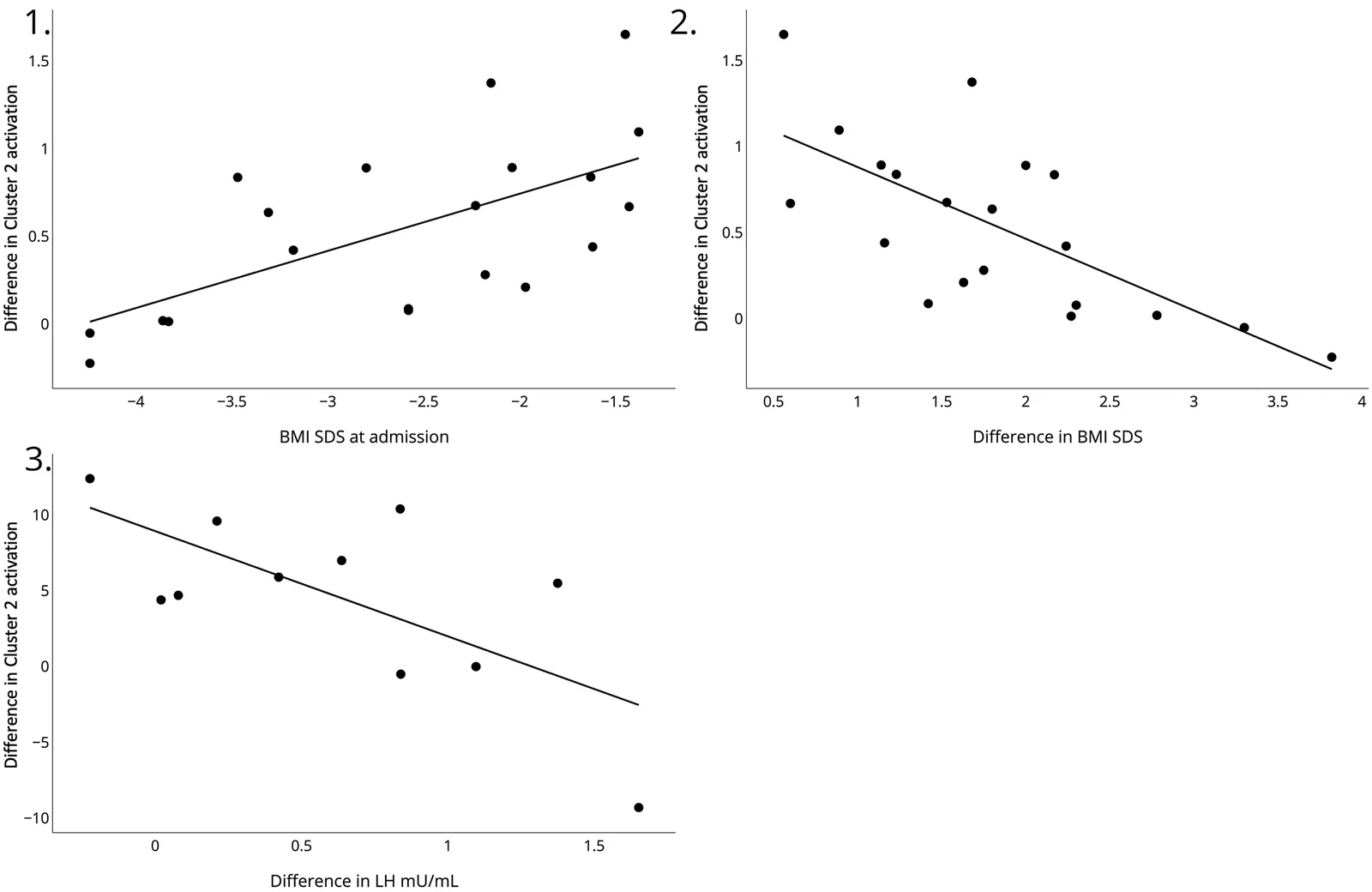
Correlation between the difference in activation (AN_Discharge_ - AN_Admission_) in Cluster 2 (Cluster covering parts of left middle and superior occipital gyrus as well as left calcarine and lingual gyrus) and **1.** BMI SDS at admission *p* = 0.003, *r* = −0.628, **2.** difference in BMI SDS *p* = 0.001, *r* = −0.694, **3.** Difference in LH (mU/mL) *p* = 0.021, *r* = −0.679.

**Fig. 5B:**
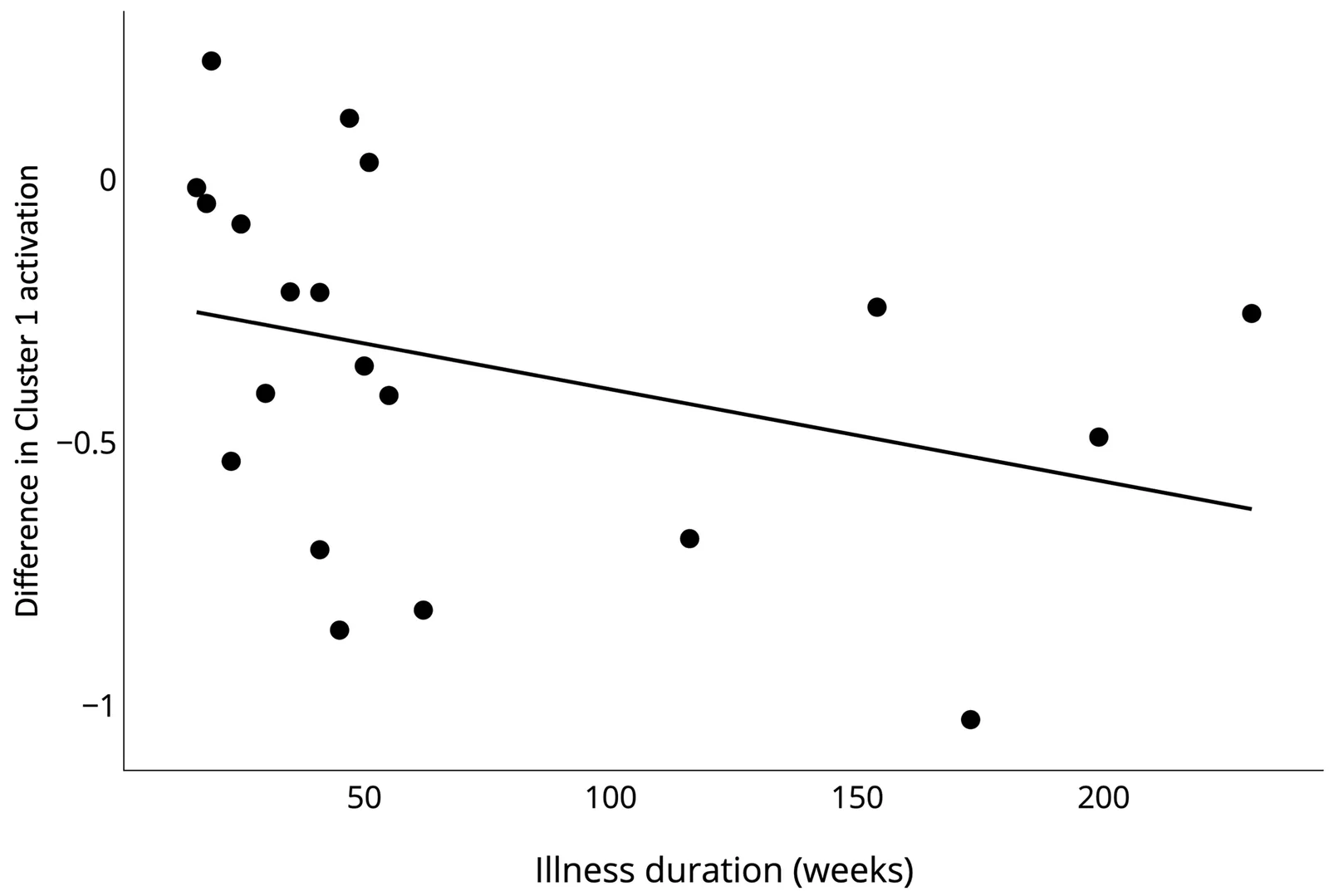
Correlation between illness duration and activation in the Cluster 1 (Cluster covering parts of left middle and inferior occipital gyrus as well as left fusiform gyrus and lingual gyrus) in AN_Admission_, *p* = 0.041, *r* = 0.46.

**Fig. 5C:**
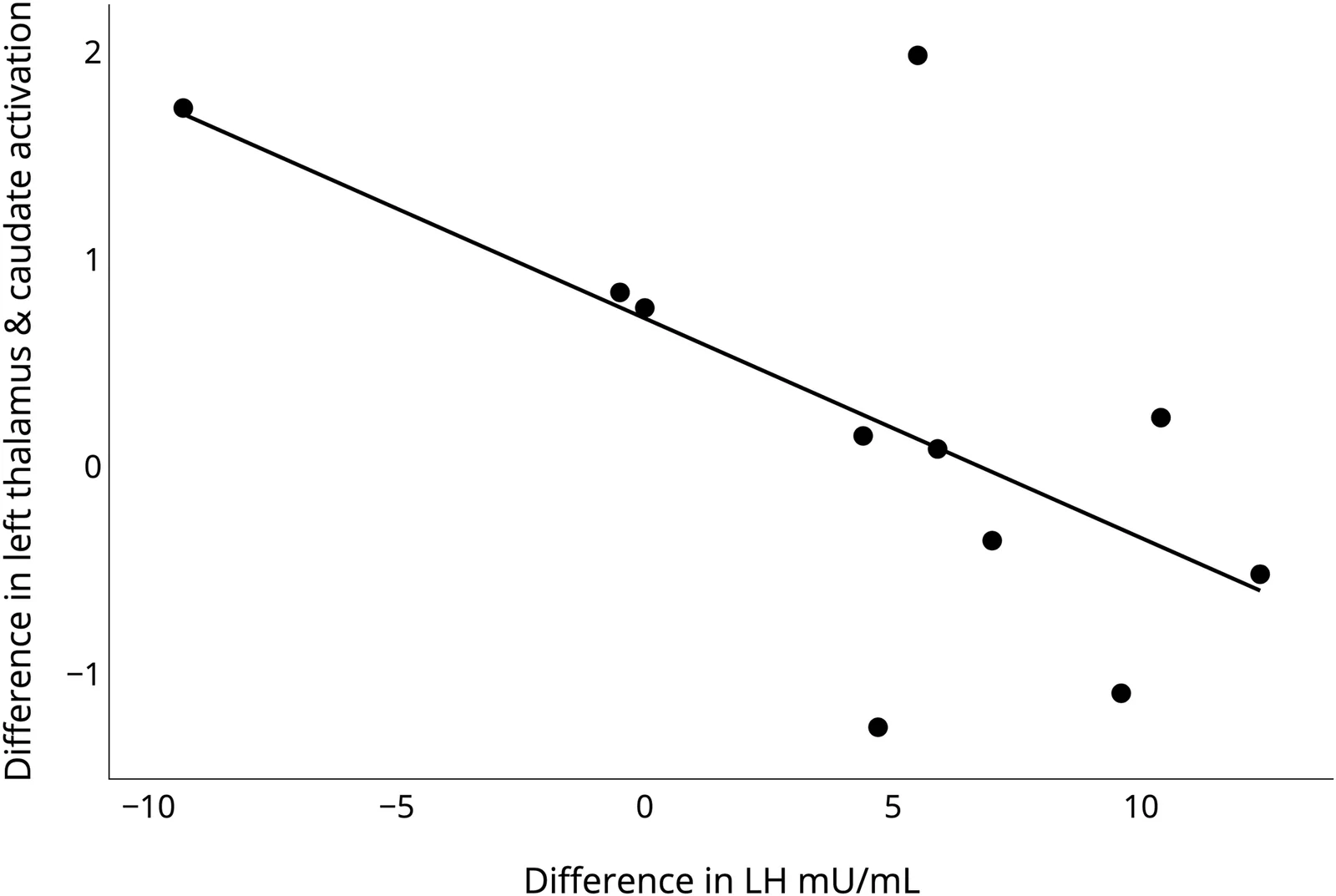
Correlation between difference in LH (mU/mL) and difference in left thalamus and caudate activation, AN_Discharge_ - AN_Admission_, *p* = 0.044, *r* = 0.616.

Brain hypoactivation based on the contrast AN_Discharge_ > AN_Admission_ showed positive correlations between activation at admission in the left lingual gyrus and delayed recall, as well as a positive correlation between the difference in the left lingual gyrus activation (AN_Discharge-_ AN_Admission_) and BMI-SDS at admission and a negative correlation between the difference in BMI SDS (Fig. 5D). Similar correlations showed in the left fusiform gyrus (Fig. 5E). Negative correlations were found between both, left lingual gyrus and fusiform gyrus activation at admission and future LH increase.

**Fig. 5D:**
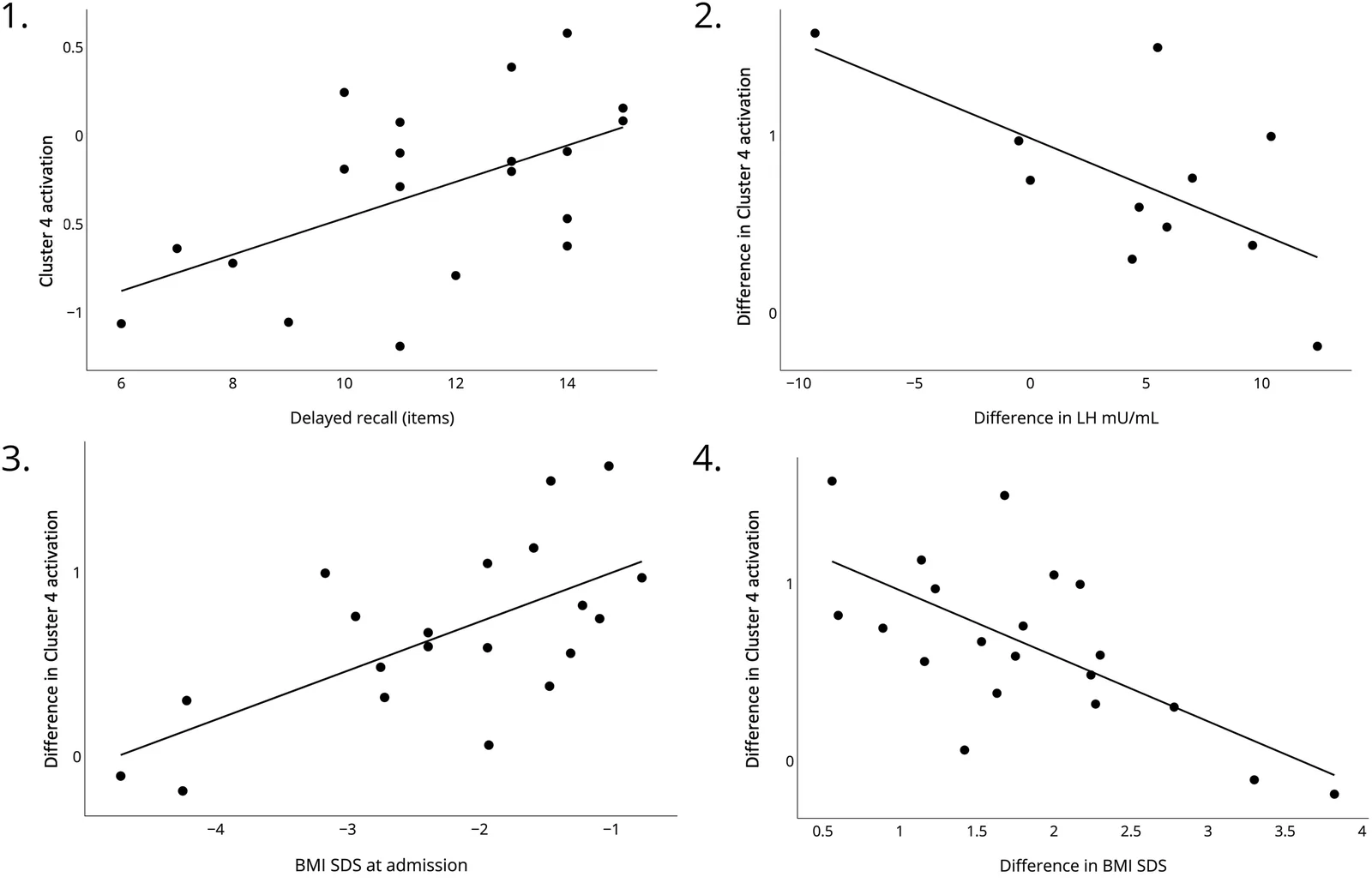
Correlation between **1.** activation in Cluster 4 (Cluster covering parts of left calcarine and lingual gyrus as well as left middle and superior occipital gyrus) and delayed recall (items) in AN_Admission_, *p* = 0.011, *r* = 0.553 and correlation between the difference in Cluster 4 activation (AN_Discharge_ - AN_Admission_) and **2.** difference in LH mU/mL, *p* = 0.035, *r* = 0.637, **3.** BMI SDS at admission *p* = 0.005, *r* = 0.602, **4.** Difference in BMI SDS *p* = 0.002, *r* = 0.659.

**Fig. 5E:**
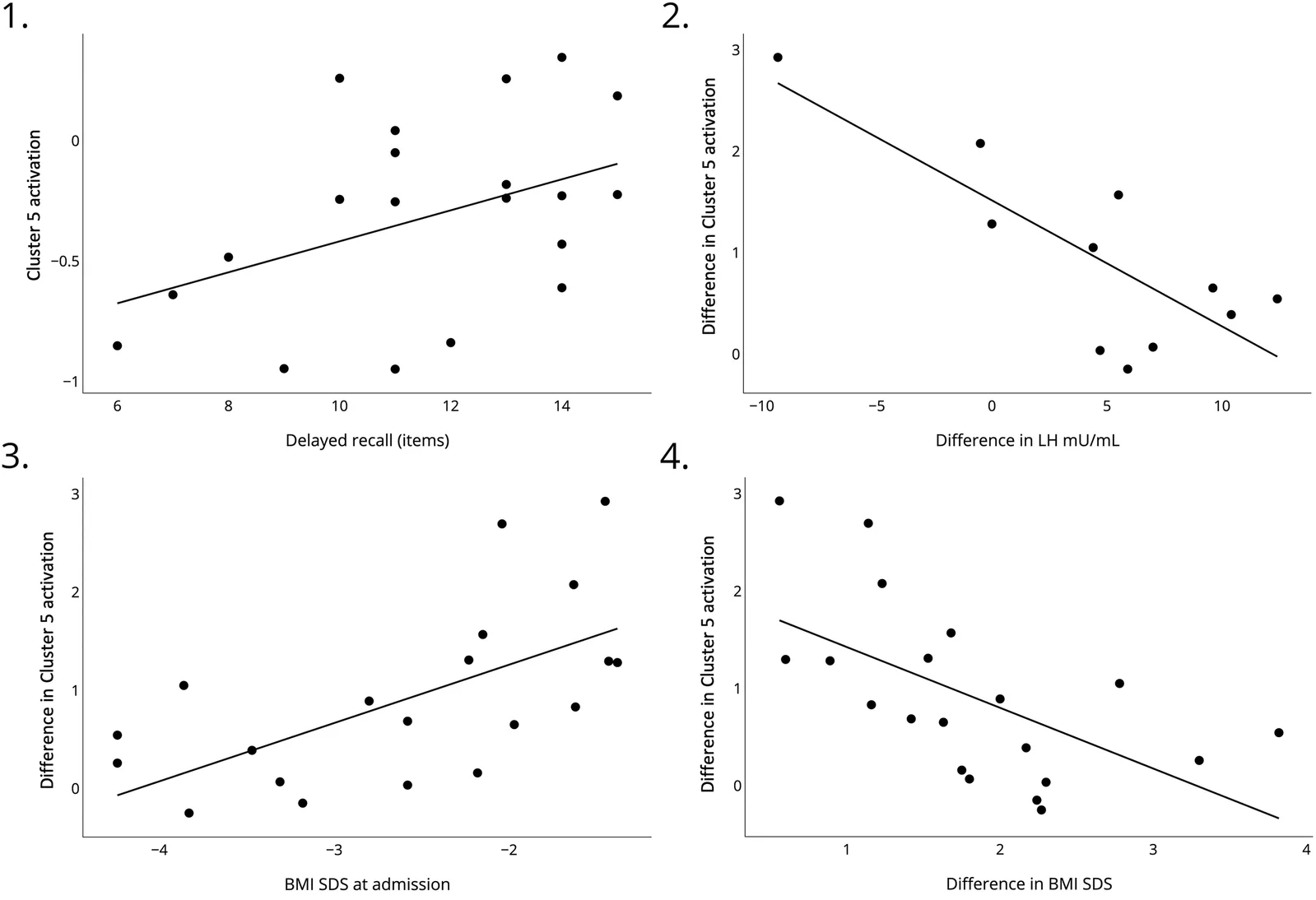
Correlation between **1.** activation in Cluster 5 (Cluster covering parts of lingual gyrus as well as fusiform gyrus while a small part of this cluster ranges into the cerebellum) and delayed recall (items) in AN_Admission_, *p* = 0.041 *r* = 0.46 and correlation between the difference in Cluster 5 activation (AN_Discharge_ - AN_Admission_) and **2.** difference in LH mU/mL, *p* = 0.004, *r* = 0.792, **3.** BMI SDS at admission *p* = 0.005, *r* = 0.599, **4.** Difference in BMI SDS *p* = 0.002, *r* = 0.659.

## 4 Discussion

To study the influence of reduced sexual hormone levels accompanying low body weight on the underlying pathomechanisms of memory and learning, we performed tasks addressing learning and memory including an implicit learning task in an MRI scanner longitudinally in adolescent patients with AN compared to HC. To our knowledge this is unprecedented in patients with AN. As hypothesized, we found reduced learning rates during acute starvation in the non-verbal RFT outside the scanner while showing improvement of in-scanner learning with short-term weight restoration in patients with AN. Alongside improvement in learning rate, hypoactivations in the superior, middle and inferior occipital gyri as well as calcarine, lingual and fusiform gyri and further Ncl. caudatus and thalamus improved with weight gain. While learning rates were linked to estradiol levels in HC with high estradiol levels, this association could only be shown on a trend level for patients with AN. Reductions in neural activation in patients were related to low BMI-SDS and those with higher future improvement in LH, thereby to more severely affected patients. These findings suggest altered neural processing during learning under acute starvation. Although no behavioural impairment was observed within the in-scanner paradigm, deficits were evident in the RFT, indicating altered learning-related processes under acute starvation. These results may contribute to understanding altered learning-related processes under acute starvation potentially responsible for difficulties of (re-)learning necessary in psychotherapy. Thus, early weight gain appears even more warranted in these patients and special regard should be given to the normalization of sexual hormones to enable successful psychotherapeutic treatment. It should be noted, however, that it remains to be shown in future studies, how learning outcomes and neural activation would further evolve after complete hormonal restitution.

Our behavioural findings of reduced implicit non-verbal learning in the RFT and the in-scanner task fit well with our hypothesis and the previous literature. Stedal et al. showed small but significant impairments in memory, working memory and visuospatial abilities in a meta-analysis including 1333 participants (665 with AN) (Stedal et al., 2021). More recent studies included implicit non-verbal learning (Wiener et al., 2023), logical memory (Terhoeven et al., 2023) and pattern recognition (Keeler et al., 2021). Verbal memory (AVLT) was not reduced in our sample, also in line with most, but not all of the literature. Dahlen et al. found worse performance for patients with AN in 29% of the studies in a review of phonological working memory in 25 studies, but no difference in 71% (Dahlén et al., 2022; Terhoeven et al., 2023). In evaluating this review, it is important to consider that different magnitude of weight loss in the individual studies is likely to have influenced these results.

The increasing learning rate in the scanner found in our patients after weight gain underscores the role of starvation-effects on learning and memory. These could be linked to metabolic factors like nutritional deficits or general undernutrition. Yet, psychological factors like exaggerated pre-occupation with weight and the phenomenon of starved patients being “locked-in” with a lack of interest in the outside world could also be another explanation for the poorer performance regarding the learning tasks upon admission. Neuroimaging studies showing a 5-10% brain volume reduction in the acute state (for a review and meta-analysis see Seitz et al., 2014) might be associated with a loss of astrocytes shown in the ABA animal model by our group (Frintrop et al., 2019). Astrocytes help to feed neurons that cannot store glycogen themselves and support synaptogenesis between neurons, which might be impaired in patients with AN (Frintrop et al., 2021).

However, these findings of brain-volume and astrocyte loss per se do not provide any information about their functional consequences and altered brain activations in patients with AN. Our novel findings of significant reductions in brain activations during implicit learning in acutely starved patients with AN in the superior, middle and inferior occipital gyri as well as calcarine, lingual and fusiform gyri and further Ncl. caudatus and thalamus help to fill that gap. Reductions in neuronal activation have been found by several authors before, including in the dorsal anterior cingulate cortex (dACC) (Geisler et al., 2017), the dorsolateral prefrontal cortex, the striatum (Ehrlich et al., 2015; Lao-Kaim et al., 2015), and the hippocampus (Keeler, 2020). One study in patients with AN also found the thalamus to be affected (Firk et al., 2015; Walton et al., 2022; Walton et al., 2022, 2022). The thalamus has been implicated in learning and decision-making in various animal models and humans including encoding of choice and specifically enhancing activity to the “go frequency” as learning proceeds (Gilad et al., 2020) and monkey magnocellular subdivisions being used for learning new information and decision making (Mitchell, 2015)). Ncl. Caudatus has been shown to be active in an associative learning model in a visual stroop-task (Chiu et al., 2017), its activity predicted perceptual learning rate for visual feature conjunction (Reavis et al., 2015) and was important for classification learning (Seger and Cincotta, 2005). Direct stimulation of the Ncl. caudatus furthermore enhanced associative learning in epilepsy patients before their operation (Bick et al., 2019). Lingual and fusiform gyrus has been associated with learning in multiple studies including verbal learning (Marcotte and Ansaldo, 2014) but also non-verbal learning. In a PET-study, Herholz et al. (2001) demonstrated face-name association learning to activate specifically lingual and fusiform gyri on both sides (Herholz et al., 2001). Furthermore, episodic encoding of pictures and words relied on this region (Köhler et al., 2000) and visual encoding of novel and familiar pictures showed reproducible activations in lingual gyrus (Machielsen et al., 2000). Davis et al. showed that in a non-verbal, longitudinal fMRI learning-task, especially perceptual memory was encoded in early visual cortex, including lingual gyrus. Conceptual memory was predicted by an activation of both lingual and fusiform gyrus amongst other regions (Davis et al., 2021). Our results regarding activations in the occipital and calcarine gyrus are in line with previous findings suggesting altered visual processing and attentional allocation in anorexia nervosa. One study on adolescents with AN has also found reduced activation in inferior, medial occipital and lingual gyri in a set-shifting task whereas there was no noticeable increase of that altered signal behavior after weight recovery (Castro-Fornieles et al., 2019). Another study on adolescent patients with AN was able to show increased activation in temporal and parietal areas including the middle occipital gyrus after weight gain in a working memory task (Castro-Fornieles et al., 2010). Previous research in adults with AN reported altered activation in occipital and posterior perceptual processing regions during cognitive flexibility tasks (Sato et al., 2013). This supports the idea that integration of sensory input is altered reflected by decreased signal behavior in acute illness which rehabilitates after weight gain. Divergent signal behavior in these regions may therefore reflect differences in perceptual processing and stimulus integration under acute starvation. The fact that those reductions of neuronal responses were mainly driven rather by trials with hits than false answers further validates our model as differential reactions to false answers and the negative feedback are ruled out, as this could have made a major impact on neural activations (Kukolja et al., 2009; Montaldi et al., 2006; Risius et al., 2019). This might be especially true in often perfectionistic patients like those with AN.

Our finding, however, ensures that the neuronal differences are disentangled and that the mentioned observation only stem from successful retrieval – and this appears to be reduced in acutely starved patients with AN.

Similar to the behavioral findings, neuronal activations increased significantly in almost the same regions following short-term weight restoration, further highlighting the potential role of nutritional restitution for brain function.

Exploratory associations found the reduced activations in these clusters to be linked to lower BMI-SDS at admission and to the increase in LH during the course of short-term weight restoration. More increase in LH during treatment infers lower starting LH values, as most patients are treated until hormonal recovery begins. Both are therefore markers of illness severity, with more severely affected patients showing even lower brain activity than their less affected co-patients. This further validates our findings and supports weight gain and hormonal restitution.

Regarding our second hypothesis of a potential role of estrogen and sexual hormones in learning and memory in AN, the results were mixed. We could find an association of in scanner learning rate with estradiol blood levels in HC, potentially linked to normal fluctuations during the menstrual cycle. This has been shown before, alongside reductions in menopause and restitution with early estradiol replacement therapy (V. Luine, 2015). Importantly, HC were not allowed to take hormonal pregnancy preventing medication to ensure that hormonal fluctuations and their influence on learning could be accurately assessed. This normal association, however, was largely lost in our patients with AN and no direct association could be found. When we performed median split into a patient group with higher and lower estradiol levels, there was a trend-level better performance at discharge in the group with higher estradiol, but no significant findings. The reason for this loss of association at admission could be due to the very low estradiol levels during starvation with lack of variability. Only after short-term weight (and hormonal) rehabilitation, a certain effect became visible. However, varying speed of weight gain, different levels of achievement of target weight sufficient for hormonal restoration and time-lag for intricate hormonal interplay to resume menses might have all contributed to adding “noise” and preventing us from finding a more distinct association. Thus, this study should be replicated with patients who resumed their menses and had a normal menstrual cycle. Estrogens have been found to be important in patients with AN in the past. Bühren et al. showed an association with verbal memory in patients with AN (Bühren et al., 2008) while Paulukat et al. from our group could show a direct association of learning rate in the novel object placement task and estradiol levels in the ABA rat model before and after starvation (Paulukat et al., 2016). This fits well with general literature on the role of estrogens in learning with facilitation of memory processing through membrane mediated mechanisms and alterations in spine density (for a review, see Luine and Frankfurt, 2012). Furthermore, Prange-Kiel and Rune could reveal estrogen-induced synaptic plasticity to be shown by an increase of spines at apical dendrites of CA1 pyramidal neurons after systemic application of estradiol to ovariectomized rats. Estradiol-induced spinogenesis in the hippocampus was also mediated by indirect mechanisms and was furthermore regulated endogenously in a paracrine manner (Prange-Kiel and Rune, 2006). Sellers et al. in their review summarized molecular signatures of rapid estrogen regulation of synaptic connectivity and their effect on cognition. Estrogen receptors can couple to intracellular signaling cascades, either directly or via the transactivation of other receptors. Subsequently, estrogen can rapidly modulate both neuronal structure in the hippocampus and the cortex (Sellers et al., 2015). Thus, estrogen plays a significant role in learning and memory which we could confirm in a subgroup of HC but only on a trend level in our patients. Significantly improving learning results following short-term weight gain which was accompanied by increases in estrogen renders further indirect support to this finding.

FSH or LH as proxies for hormonal restitution did not yield any significant results apart from relatively lower LH at admission, allowing relatively greater LH normalization during refeeding, being associated with further reduced neuronal learning activation. One can speculate that LH might also be important for restoring learning-associated neural activity, however, lower LH could also be a marker of a longer way to general somatic and neurological restitution. Thus, although hormonal replacement with estradiol in patients with AN is proposed to be taken into consideration not only in the German S3 treatment guidelines for AN but also the NICE-guidelines (Wilson and Shafran, 2005)for the compensation of accompanying osteoporosis for amenorrhea longer than one year (Herpertz et al., 2019), more studies are needed to prove a link between estradiol and learning that would have immediate therapeutic consequences by extending this indication of estradiol substitution to learning difficulties. In any case, swift and extensive enough weight gain to regain menstrual cycle and thus adequate endogenous estrogen levels remain already today the main consequences of our results.

## 5 Strengths and limitations

This study's strengths are the inclusion of adolescent patients, which tend to have a shorter duration of illness and thus less chance of chronification. This might give insight into an earlier stage of the illness with less compensatory or failed treatment effects altering the neural picture. Furthermore, the inventive design of using a well-established dynamic learning paradigm together with hormonal measurements in the same participants has not been used before in this combination. Lastly, our longitudinal design allowed to separate potentially starvation-induced state-effects at admission from remaining trait effects after short-term weight rehabilitation in the same patients.

The biggest limiting factor is the modest sample size. Although we did find significant differences during acute starvation, we might have missed information about involvement of additional brain regions, more delicate sex-hormone associations or small remaining alterations after short-term weight recovery. Larger studies are necessary to clarify this matter. Furthermore, we only looked at short-term weight recovery. Long-term weight recovery and prospective studies might unravel a potentially prognostic role of impaired learning and memory for clinical prognosis.

## 6 Conclusion

We found reduced implicit learning rates and associated reduced brain activations in acutely starved adolescents with AN in in the superior, middle and inferior occipital gyri as well as calcarine, lingual and fusiform gyri and further Ncl. caudatus and thalamus. Both learning and brain activations increased significantly with short-term weight restoration and associated hormonal restitution. However, a direct link between learning rate and estradiol levels could only be shown for HC with high estradiol but not in patients with AN at either timepoint. As a substantial number of patients concluded treatment while still being amenorrheic, likely due to the limited inpatient time that is insufficient for the complete re-establishment of a regular menstrual cycle, a replication of the investigation after regaining the menstrual cycle certainly could confirm and expand our results. These results add to our knowledge of underlying brain pathophysiology of AN in general and learning and memory in these patients in particular. Impaired neural activation and reduced learning in the acute state might help explain difficulties in (re-)learning and psychotherapy often encountered in these patients and further reinforce the need for rapid weight and hormonal restitution in patients with AN.

## CRediT authorship contribution statement

**Kimberly Büttgen:** Writing – original draft, Validation, Methodology, Investigation, Formal analysis, Data curation. **Jana Goedecke:** Writing – review & editing, Visualization, Validation, Software, Formal analysis, Data curation. **Lukas Stanetzky:** Writing – review & editing, Investigation, Data curation. **Brigitte Dahmen:** Writing – review & editing, Supervision. **Beate Herpertz-Dahlmann:** Writing – review & editing, Supervision, Resources, Project administration, Methodology, Funding acquisition, Conceptualization. **Kerstin Konrad:** Writing – review & editing, Supervision, Resources, Methodology, Conceptualization. **Oezguer A Onur:** Writing – review & editing, Validation, Software, Resources, Methodology, Formal analysis, Conceptualization. **Jochen Seitz:** Writing – review & editing, Validation, Supervision, Software, Resources, Project administration, Methodology, Funding acquisition, Formal analysis, Conceptualization.

## Declaration of competing interest

The authors declare that they have no known competing financial interests or personal relationships that could have appeared to influence the work reported in this paper.

## Data Availability

The authors do not have permission to share data.
