# Supplementary material for: Neural correlates and sex hormonal associations of impaired feedback-learning in adolescent patients with anorexia nervosa - a longitudinal fMRI study
Source: Neuroimage Rep. 2026 Sep 30;6(4):100408. doi: 10.1016/j.ynirp.2026.100408 (PMC13654061; doi:10.1016/j.ynirp.2026.100408)
Supplement: Supplementary file 1 [file mmc1.docx]

Supplementary

Supplemental Figure 1

Mean betas of the clusters separated for “Hits” and “False” with standard deviation. 1.1 refers to the left fusiform gyrus cluster in the HC > AN_Admission_ contrast, accordingly 1.2 refers to the left lingual gyrus cluster, 1.3 refers so the left thalamus and caudate cluster. 2.1 refers to the left lingual gyrus cluster in the AN_Discharge_ > AN_Admission_ contrast, 2.2 refers to the left fusiform cluster.

Supplemental Figure 2a

“Correlation between performance % with VLMT in ANAdmission. VLMT Cycle 1–5 (%) refers to the percentage of correctly recalled words across the first five learning trials of the Verbal Learning and Memory Test (VLMT), whereas Cycle 7 (%) reflects delayed recall performance.“

Supplemental Figure 2b

Learning rate per cycle per group, calculate as performance in cycle 6 - performance in cycle 1 with standard error. *significant **highly significant in student’s T-Test.

Supplemental Figure 3

Scatter plot for AN_Admission_ and AN_Discharge_ with high estradiol and learning rate %.
