# Supplementary material for: Neural correlates and sex hormonal associations of impaired feedback-learning in adolescent patients with anorexia nervosa - a longitudinal fMRI study
Source: Neuroimage Rep. 2026 Sep 30;6(4):100408. doi: 10.1016/j.ynirp.2026.100408 (PMC13654061; doi:10.1016/j.ynirp.2026.100408)
Supplement: Supplementary file 2 [file mmc2.docx]

**Supplementary Table 1. Anatomical labelling of the clusters**

| **Cluster** | **Peak Structure aal label** | **Structure aal label (percentage)** |
| --- | --- | --- |
| **1** | Peak 1: Cerebelum_Crus1_L | Occipital_Mid_L(35) Occipital_Inf_L(28) Fusiform_L(20) Cerebelum_Crus1_L(8) Lingual_L(4) Cerebelum_6_L(1) |
|  | Peak 2: Occipital_Inf_L | Temporal_Inf_L(34) Temporal_Mid_L(30) Occipital_Inf_L(21) Occipital_Mid_L(10) Angular_L(2) Fusiform_L(1) |
| **2** | Peak 1: Lingual_R | Occipital_Mid_L(39) Occipital_Sup_L(21) Calcarine_L(14) Lingual_R(10) Lingual_L(4) Vermis_6(4) Calcarine_R(3) |
| **3** | Peak 1: Thalamus_L | Thalamus_L(52) Caudate_L(11) |
| **4** | Peak 1: Calcarine_L | Calcarine_L(32) Occipital_Mid_L(24) Occipital_Sup_L(19) Calcarine_R(13) Lingual_L(2) |
|  | Peak 2: Occipital_Mid_L | Occipital_Mid_L(100) |
| **5** | Peak 1: Cerebelum_Crus1_L | Lingual_L(55) Cerebelum_Crus1_L(31) Cerebelum_6_L(10) Fusiform_L(4) |
